# Supplementary material for: Are carbon emissions trading and green financial instruments synergistic? -Comprehensive quantitative research based on content analysis
Source: PLoS One. 2024 Mar 7;19(3):e0298601. doi: 10.1371/journal.pone.0298601 (PMC10919601; doi:10.1371/journal.pone.0298601)
Supplement: S1 Data — (ZIP) [file pone.0298601.s001.zip › Data/地级市Z_F工作报告.zip!/║■▒▒/╢⌡╓▌╩╨/└┤╫╘╣1⁄2╓┌║┼í╛┬φ┐╦╩2╛▌═°í┐_2007.docx]

一、2006年工作回顾

　　2006年是鄂州经济社会发展取得显著成就的一年。在省委省政府和市委的正确领导下，我们紧紧依靠全市各级干部和广大人民群众，坚持以科学发展观统领工作全局，围绕“努力建设武汉城市圈发展最快中等城市”的总目标，发展新项目、建设新农村、营造新环境，经济社会呈现出持续、快速、协调、健康发展的良好态势，全面或超额完成了市五届人大三次会议确定的各项目标任务，实现了“十一五”的开门红。

　　（一）发展速度明显加快。全年完成地区生产总值168.3亿元，同比增长13.1%；完成全社会固定资产投资72.1亿元，增长30.6%；规模以上工业完成增加值70.9亿元，增长25.5%；实现财政收入16.6亿元，增长24.9%，其中地方一般预算收入达到6.5亿元，增长31.2%；实现社会消费品零售总额71.6亿元，增长15.2%；招商引资总额达到3.7亿美元，同比增长28%；实际利用外资6633万美元，是2005年的2.3倍；完成外贸出口海关数7683万美元，增长41.1%。

　　（二）发展质量明显提高。结构日益改善。产业结构更趋优化，三次产业比由去年的16.2：47.3：36.5变为14.9：50.2：34.9；企业结构得到改善，规模以上工业企业达到237家，同比增加38家。共投入6608万元，对25家重点企业进行改制，除2家等待政策性破产外，列入两改计划的131家企业已基本完成改制任务；名牌创建初见成效，共申报成功湖北名牌7个，中国名牌1个，实现争创中国名牌零的突破；12件注册商标获湖北省著名商标称号，“鄂州武昌鱼”原产地证明商标在国家工商总局成功注册。效益大幅提高。规模以上工业企业实现利税总额16亿元，增长42.7%；工业经济效益指数达到183.6，同比提高17.4个百分点。城乡居民收入增长较快。城镇居民人均可支配收入达到9013元，增长10.7%；农民人均纯收入达到3800元，增长8.7%。

　　（三）发展后劲明显增强。开工项目和储备项目增多。全年有鄂钢冷轧薄板、鄂钢宽厚板、中非膨润土加工、团结激光、瑞合铁路配件等13个亿元以上项目开工建设，有51个千万元以上项目签约，项目建设态势之好前所未有。规划和土地储备得到加强。明确了“一主三新十特”的城镇发展体系。聘请了全国一流规划设计院对葛店开发区、花湖开发区、红莲湖旅游度假区进行了科学规划。全年共批回建设用地8125亩，是前三年的总和。基础设施日益改善。城市基础设施建设投入力度加大，旭光大道、鄂钢跨线桥连接路、球团矿立交桥等6个项目已竣工通车，武昌大道东段延伸、寿昌大道、沿江大道等6项续建工程正在抓紧施工。交通建设完成投资2.2亿元，樊口大桥进入全面施工，关葛一级公路（高新大道）、庙岭立交桥建设稳步推进，樊口大桥接线工程完成招标。鄂州火车站升级为二级站，华容火车站竣工验收。房地产市场健康发展；园区基础设施建设投入达到3.29亿元，创历史最高水平。

　　（四）发展环境明显改善。行政环境逐步优化。制度建设力度加大，行政执法行为进一步规范，实行了收费明白卡制度，依法行政水平逐步提高。开展了发展环境万人评议活动，市直各部门服务发展的意识进一步增强。政务事务公开、经济责任审计、行政效能监察、打击商业贿赂等源头治腐工作稳步推进。治安环境有所改善。普法教育深入推进，社会治安综合治理得到加强，强装强卸、堵门堵路等违法行为明显减少，重大恶性案件得到有效遏制。社会环境更加和谐。大力整顿市场秩序，工商行政管理、质量技术监督和食品、药品、烟草市场监管进一步加强。通过对电力的合理安排与调度，满足了工农业生产和人民生活的需要。落实了安全生产“一票否决”制度，安全生产保持了平稳态势。妥善解决了程潮铁矿地质灾害问题。信访工作得到加强，市领导实行包案下访，亲自督办信访件的落实，有力地促进了社会和谐稳定。信用环境明显好转。进一步完善了中小企业贷款信用担保体系，担保公司增资扩股顺利完成。金融机构年末存款余额114.5亿元，比年初增加16.3亿元；贷款余额72.4亿元，比年初增加7.2亿元。

　　（五）发展格局明显优化。社会事业全面发展。基础教育稳步提高，中职教育加快发展，高中招生实现城乡统一划线，高考再创佳绩；文化事业日益繁荣，重大科技项目实施取得新突破；未成年人思想道德教育得到加强，非物质文化遗产保护取得进展，“农民体育健身工程”全面展开；经多方努力争取，我市整体纳入了全国新型农村合作医疗试点，80%以上的农民开始享受合作医疗；投资870万元，对乡镇卫生院实施全面改造；进一步完善了重大疾病和疫情的防控体系；社会保障体系逐步完善，调高了低保标准，在全省地级市中率先启动农村低保。企业离退休人员增加养老金、国有困难企业退休人员实行医保、机关事业单位工资改革、规范津补贴工作稳步推进。筹资开发了600个城镇公益岗位。下岗失业人员小额担保贷款稳步开展，全年发放小额担保贷款360万元。城乡困难群体的社会救助体系逐步完善。新农村建设开局良好。共投入建设资金8000多万元，实施农业项目190个。农村经济发展较快，粮食、水产、蔬菜、畜牧产量全面增长。投入1950万元，解决了5万人的饮水安全问题；投入5000多万元对17座排涝55千瓦、33座抗旱30千瓦的泵站进行了改造。投入1000万元建设农村公路100公里，投入570万元对30个重点贫困村实施整村推进扶贫开发。通过实施“阳光工程”、“雨露计划”，共培训农村劳动力1.8万人，转移就业率达96%。粮食购销政策得到较好落实，供销、农资市场进一步繁荣。乡镇综合配套改革基本完成，村级组织办公活动场所建设进展顺利。经济建设与人口增长、生态环境保护日趋协调。人口自然增长率控制在5‰以内。环境保护得到加强，投入2200万元完成了洋澜湖综合治理一期工程和五丈港改造工程，顺利通过国家生态示范区创建验收。进一步规范了矿产资源管理工作，无证勘查和乱采滥挖等违法采矿行为得到遏制。万元生产总值综合能耗下降4%。

　　与此同时，广播电视、民族宗教、外事侨务、对台、物价、统计、住房公积金管理、保险、邮政、电信、石油石化、档案、方志、新闻出版、人防、防震减灾、消防、老龄、气象、农机和国防教育、民兵建设等工作都取得了新的成绩。

　　在此，需要向各位代表专题报告的是，五届人大三次会议上，市政府向全市人民承诺的“十件实事”已圆满完成。“十件实事”共筹措资金11405万元，占计划筹资的154%，直接受益人口达21万多人。通过“十件实事”的实施，进一步密切了党和政府与人民群众的联系，受到全社会的普遍赞誉。还需要向各位代表报告的是，2006年全市纳税千万元以上的企业是鄂钢、鄂州电厂、程潮铁矿、供电公司、吴城钢铁公司、烟草公司、世纪新峰公司、陈盛铁矿、波尔亚太公司、华中重机、鸿泰钢铁公司。

　　各位代表，过去一年的成绩来之不易，这是市委正确决策和坚强领导的结果，是人大依法监督和支持、政协参政议政和民主监督的结果，是全市人民团结奋斗的结果。借此机会，我谨代表市人民政府向在各个岗位上辛勤工作的广大干部群众，向各位人大代表、政协委员，向各民主党派、工商联、无党派人士、人民团体和社会各界人士，向为鄂州发展做出贡献的各位老同志，向驻军指战员、武警官兵以及所有来鄂州的投资者、劳动者和海内外朋友表示崇高的敬意和衷心的感谢！

　　各位代表，当前我市在加快发展中还面临许多困难和矛盾，政府工作还存在一些不足和差距：一是经济结构调整和增长方式转变任务艰巨。产业过于单一，没有形成产业链，自主创新能力不强。现代服务业比重偏低，民营经济规模不大，实力不强。二是县域经济发展较慢。县（区）域经济发展低于全省县区平均水平，经济增长点不多，财政较为困难；园区建设滞后，经济建设受到发展平台的制约。三是和谐鄂州建设任务较重。社会保障体系仍不健全，就业再就业压力较大，部分下岗职工、城镇低收入居民和贫困地区群众的生活还比较困难。资源环境面临较大压力。社会治安状况仍待改善。四是政府自身建设仍需加强。政府的工作效能和依法行政水平仍待提高，廉政建设、作风建设需进一步加强。对这些困难和问题，我们将高度重视，采取得力措施，认真加以解决。

　　二、2007年工作安排

　　今年政府工作的指导思想是：认真贯彻党的十六届六中全会精神，以科学发展观统领全局，围绕建设武汉城市圈发展最快中等城市的战略决策，围绕解决人民群众最关心、最直接、最现实利益问题的基本要求，围绕建设创业鄂州、宜居鄂州、和谐鄂州的总体目标，坚持“三新”思路，推进“四城”创建，着力调整经济结构和转变增长方式，着力推进改革开放和自主创新，着力加强资源节约和环境保护，着力推进社会发展和解决民生问题，努力实现经济社会又好又快地发展。主要预期目标是：地区生产总值188亿元，增长12.5%；财政收入19.6亿元，增长18%；一般预算收入7.4亿元，增长15%；全社会固定资产投资86亿元，增长20%；社会消费品零售总额81亿元，增长14%；外贸出口8600万美元，增长12%；实际利用外资7400万美元，增长10%；招商引资总额30亿元，增长20%；城镇居民人均可支配收入9911元，增长10%；农民人均纯收入4100元，增长6%；城镇新增就业岗位2万个以上，城镇登记失业率控制在4.5%以内；人口自然增长率控制在5‰以内，万元GDP综合能耗下降4%。

　　上述目标的提出是在分析国家宏观经济政策、研究鄂州的经济基础和发展潜力后确定的，主要指标均达到和超过全省平均水平，只要全市上下团结一心，奋发努力，是完全可以实现的。

　　（一）坚持不懈地推进项目建设，着力夯实发展基础

　　一是集中精力加快重点项目建设。全市安排固定资产投资86亿元，投资千万元以上重点项目60个（其中亿元以上项目16个）。重点支持鄂钢宽厚板项目、鄂钢辅业外迁、重型机械扩改项目、鄂州电厂二期项目、宝业住宅产业化项目、华中重机H钢等工业项目；支持恒大金碧天下、省水上训练竞赛基地等社会发展项目。对这些投资强度大、带动能力强的重点项目，在用地、电力、资金等生产要素的配置上予以倾斜和及时协调，确保项目建设的施工进度和建设质量。

　　二是集中精力扩大招商引资规模。大力推进专班招商，创新招商方式、拓宽招商领域、建立招商基地，力争对重点地区的招商取得实质性进展；大力推进园区招商，吸引有实力的投资者落户；大力推进产业招商，围绕钢铁、化工、医药、农产品深加工编制项目，从单个项目招商变为整个产业招商。紧盯世界500强、国内500强和民营500强企业，力争在引进重大项目上取得突破。积极支持企业扩大生产能力和进行技术改造，增加投资规模。继续抢抓中部崛起和武汉城市圈建设的战略机遇，在新农村建设、公共服务、资源环境、基础设施等领域，加大向上争取项目资金的力度。

　　三是集中精力完善园区基础设施。重点抓好葛店开发区高新大道、葛庙路扩宽改造等工程；鄂州经济开发区的蒲团路、污水处理厂等工程；花湖经济开发区的花湖大道排水管网建设以及花杨路基础工程；红莲湖度假区的红莲大道扩宽改造、金碧大道、庙岭立交桥等工程；研究政策措施，盘活小桥都市工业园；抓住武汉80万吨乙烯下游产品开发和鄂钢附属企业外迁的机遇，规划建设好塑料工业园和钢铁深加工产业园。

　　（二）坚持不懈地推进结构调整，整体提升产业竞争力

　　一是以规模化为方向调优传统产业。全力争取鄂州球团厂二期工程早日开工，支持鄂钢、吴城公司、鸿泰钢铁系列技改项目，切实提升冶金行业产品档次；加快实施世纪新峰年产500万吨水泥生产项目，开发新型墙体材料、新型装饰装修材料和无机非金属新材料；支持华中重机、鄂重、富升锻压、鄂丰模具等企业加大技改力度，开发环保机械设备、大型卷板机、螺旋压力机、塑料精密模具等产品，提升机械制造业的竞争能力；支持新爱迪鞋业、多佳品牌服饰等项目建设，提升轻工纺织服装行业的整体素质；继续实施中小企业“成长工程”，力争30家企业发展成为规模以上企业，6家企业发展成为中型企业。

　　二是以板块化为方向做强新兴产业。全力争取国家生物医药产业基地落户葛店，大力引进带动力、辐射力强的生物医药项目，做强生物技术及新医药板块；开发高性能金属材料、功能陶瓷材料、燃料电池及相关材料、医用材料等新型产品，做强新材料板块；争取与省信息产业厅共建葛店信息产业园，支持莲花山视觉研究院发展，做强电子信息板块；充分发挥“鄂州武昌鱼”原产地证明商标品牌优势和水产品加工技术优势，推进农产品加工增值，做强农产品加工板块。力争生物医药、新型材料、电子信息和农产品加工四大产业增加值提高20－30%。

　　三是以集约化为方向发展特色产业。对燕矶金刚石刀具、白浒镇泵业、葛店生物医药、梁子湖非金属矿加工、华容纺织服装、段店医药软管等初具雏型的特色产业进行重点扶持和积极引导，延长产业链，做大规模。以重大项目、重点企业为依托，推动钢材深加工产业、石化下游产业、电子信息产业集群发展。将工业布局与小城镇和新农村建设有机结合，形成“一镇一业”、“一村一品”的特色经济。

　　四是以市场化为方向加快发展现代服务业。加快发展鄂东物流中心、开发区纺织冶金建材、长岭水产品等专业批发市场。加快红莲湖旅游度假区和梁子湖（岛）旅游区建设，深度开发莲花山、西山旅游资源，大力发展乡村旅游，加大旅游商品开发力度，打造具有鄂州特色的精品名牌，积极筹办红莲湖旅游高层论坛和第八届梁子湖捕鱼旅游节。利用沿江深水岸线资源，大力发展港口物流业。加强通信基础设施建设，推进城乡信息化进程。积极发展金融、保险、设计咨询及中介组织等新兴服务业，加快发展连锁经营、物流配送、电子商务等现代业态。

　　（三）坚持不懈地发展现代农业，加快新农村建设步伐

　　一是稳步推进“六个一体化”。积极探索以工促农、以城带乡的新机制，启动“六个一体化”的规划和实施方案编制工作。科学编制镇村规划，继续推进“百镇千村”工程，合理确定中心村的布点和数量，制定优惠政策鼓励引导农民向集镇和中心村集聚。加快城乡公共交通一体化和农村市场体系建设；实施“万村千乡”工程，建立完善农村消费维权体系，为农民生产生活创造方便安全消费环境；继续实施“阳光工程”和“雨露计划”，加强农民技能培训，加快转移农村富余劳动力；加快实施一村一名大学生计划，培养科技带头人、农民企业家和致富领路人。

　　二是加快发展现代农业。突出建设产业推动型农业，突破性发展农产品精深加工，以加工促调整、带基地。扎实推进50万亩优质稻板块、50万亩无公害水产板块、20万亩无公害蔬菜板块、20个畜禽小区、10万亩林果基地建设。突出建设现代装备型农业，以提高主要农作物全程机械化水平为重点，加快“以机代牛”步伐，加快设施农业、生态观光休闲型农业建设。突出建设科技先导型农业，大力推广无公害种养技术、良种和模式化栽培技术和农村能源技术，努力实现主要农产品的标准化生产。

　　三是努力改善农村条件。筹资6000万元对花马湖（洋澜湖）等泵站进行改造，筹资2000万元对马龙口等水库进行整险加固，筹资3000万元加固圩堤和磨刀矶等50座涵闸，筹资1000万元建设100公里乡村公路。积极推进有线电视村村通工程。加快文化、体育、卫生等基础设施建设。继续开展整村推进扶贫开发工作。加快推进以“一建三改”（建池、改厕、改厨、改圈）为重点的生态家园建设和以土地整理为主要内容的高产农田项目建设，全面改善农民生产和生活条件。

　　（四）坚持不懈地推进改革开放，不断增强发展动力

　　一是深化财税体制改革。坚持依法治税，加强税费征管，加快税收信息化进程，提高税收征管质量和效率。认真贯彻落实《湖北省政府非税收入管理办法》，对市直执收执罚单位全面启用新的非税收入征管模式，进一步规范非税收入分配秩序，从源头上预防和治理腐败。加强部门预算和专项资金管理，规范政府采购行为。继续推进区乡财政管理体制改革，合理划分区乡政府的事权及财政保障责任，完善转移支付制度。

　　二是深化农村综合改革。巩固乡镇事业单位改革成果，进一步完善竞争上岗、人员分流安置制度，制定“以钱养事”标准，提高农村公益服务质量和效率。千方百计化解乡村债务，巩固税费改革成果。做好新时期农民负担监督管理工作。进一步深化农村义务教育管理体制改革，建立义务教育经费保障机制，落实农村中小学公用经费。稳步推进集体林权制度改革，真正将林地使用权和林木所有权确权到户，保障农民的合法权益。

　　三是深化国有企业改革。对已经改制的企业，认真做好规范完善工作，健全法人治理结构，转换经营机制，强化内部管理。对没有改制的企业，制定可行措施，有重点分步骤地逐步解决。健全和完善国有资产监督管理体制，不断提高监管水平。

　　四是深化投资体制改革。引导、鼓励社会资本进入基础设施、公用事业等领域。进一步落实企业投资自主权，逐步放宽对投资、技改的审批和审核。改革政府投资管理体制，完善投资主体和项目责任制，从根本上改变投资决策无人问责的状况。积极探索利用资本市场和各种金融工具创新政府融资方式，提高资金运作能力。

　　五是努力扩大对外开放。保持外贸出口持续稳定增长，在巩固和发展现有出口企业的同时，不断培植和引进外贸出口企业和项目；优化出口商品结构，扩大高新技术、机电及农产品的出口，把葛店开发区建成外贸出口的重要基地。鼓励和支持企业开展对外工程承包与劳务合作，创新合作方式，扩大合作规模。加大对外人才交流力度，大胆引进优秀人才。进一步拓宽与国外友好城市的交流渠道，开展多领域合作。

　　（五）坚持不懈地推进城镇建设，加快城乡一体化进程

　　一是强力推进县域经济发展。进一步落实加快县域经济发展的各项政策，依法扩大三区的经济自主权、事务统筹权和社会管理权；探索适合三区经济发展的重点领域和方向，鄂城区大力发展资源深度开发和加工项目，华容区全面推进与东湖、葛店开发区的融合，主动承接产业转移和发展配套项目，梁子湖区大力发展非金属矿加工和旅游开发项目；支持和鼓励区、中心镇创办产业园，多途径筹集资金，改善园区水电路等基础设施，增强项目吸引力和承载能力；坚持县域经济以民营为主，消除制约民营经济发展的观念、政策和体制障碍，支持民间资本进入法律法规未禁止的领域，营造良好的创业环境，激发民间创业热情。

　　二是加强规划的指导和调控。按照建设“创业”、“宜居”和“发展最快”城市的总体要求，根据经济流向、发展潜力和城乡协调、区域联动的思路编制城镇体系规划。启动主城区总体规划的修编工作，抓紧做好红莲湖旅游新城、葛华科技新城和花湖工贸新城详规的编制工作。进一步强化规划的“龙头、刚性、法定”地位，加强规划的实施和监管。所有建设用地必须符合规划要求，坚决拆除各类违章建筑。

　　三是加快实施城镇建设十大工程。按照扩展骨架、提升品位、完善功能、改善环境的要求，投资15亿元加快实施十大基础设施建设工程：1、社会发展基础设施项目9个，投资8470万元；2、农业基础设施项目8个，投资20867万元；3、电力基础设施项目5个，投资11050万元；4、交通基础设施项目5个，投资17218万元；5、主城区城建基础设施项目8个，投资28905万元；6、通信基础设施项目4个，投资8250万元；7、葛店开发区基础设施项目4个，投资32000万元；8、鄂州开发区基础设施项目5个，投资7700万元；9、花湖开发区基础设施项目5个，投资6320万元；10、红莲湖旅游度假区基础设施项目5个，投资9220万元。

　　四是不断提高城镇管理水平。改革完善城市管理体制，建立城市管理的长效机制，形成全社会共同参与城市管理的局面。集中开展以交通秩序和占道经营为重点的综合整治，创建若干个城管达标示范社区和路段。提高社区管理水平，加强对公益岗位中从事“门前三包”、小区保洁、市容管理人员的管理，实行街道社区和城管部门的双向考核，明确责任，提高效率，使环境整治工作由单位庭院向居民家庭和楼道延伸，由大街马路向背街小巷延伸。加强物业管理，重点解决好水、电直供问题。区分不同情况，采取不同方式，稳步推进城中村改造。将城市管理工作纳入部门年度工作责任目标，严格进行考核。

　　（六）坚持不懈地推进增长方式转变，努力实现又好又快的发展

　　一是大力推进资源节约和环境保护。重点抓好钢铁、电力、建材等行业以及年耗10万吨标煤以上用能大户的节能降耗工作。执行建筑节能设计标准，发展节能省地型建筑。加强土地调控管理，抓紧完善和严格执行节约集约用地标准，坚决制止违法违规用地行为，提高土地利用效率和集约化程度。整顿和规范矿产资源开发秩序。坚决依法关闭不符合国家产业政策、严重浪费资源和污染环境的企业，加快淘汰落后的高耗能、高耗水、高耗材的工艺、设备和产品。对立窑水泥企业按计划关停改造。加大环保执法力度，从源头上控制环境污染。加强重点区域的水资源保护和水污染、空气污染、噪音污染治理。严格执行排放总量控制、排污许可、限期治理和建设项目环境影响评价制度，完善环境监测预警体系与环境监察体系。

　　二是加快发展循环经济。在冶金、建材等重点行业，矿产资源综合利用等重点领域，组织实施一批减量化、资源化、再利用示范工程项目；依托鄂钢、鄂州电厂等骨干企业的余热和副产品，发展循环工业示范项目。推广清洁生产和施工新模式，运用市场手段促进资源循环利用。推广先进适用的开采技术、工艺和设备，提高采矿回采率、选矿和冶炼回收率，大力推进尾矿、废矿综合利用，提高资源综合回收利用率。

　　三是加强自主创新能力建设。坚持把自主创新的着力点放在消化吸收再创新上，依托武汉高校院所引进科技项目和人才，建立产学研结合的创新体系。充分发挥财政资金的引导作用，鼓励企业加大科技投入，对争创国家级技术中心的企业和中国名牌、中国驰名商标的企业给予奖励。加大知识产权保护力度，指导和支持企业开发专利技术，形成一批拥有自主知识产权的专利产品。推行科技人员岗位工资制、项目工资制、骨干科技人员年薪制以及股权、期权奖励等多种形式的激励机制，充分调动科技人员的积极性和创造性。

　　（七）坚持不懈地推进环境建设，努力提升鄂州形象

　　一是营造良好的创业环境。实行行政事业性收费一卡制，清理收费项目，降低收费标准，取消不合理收费。加强行政许可配套制度建设，规范行政审批。试行投资商便利卡制度，在全社会倡导尊重投资者、企业家和纳税人的良好风气。深入开展创建诚信企业活动，督促企业以良好的信誉赢得金融机构长期性的信贷支持。加快民营企业、中小企业信用担保体系建设，鼓励社会资本、民间资本进入中小企业信用担保领域，引导民营企业按行业、以会员制方式组建互联、互保、互助性的担保机构，逐步解决民营企业、个人创业融资困难的问题。重视进城务工人员的劳动保障和生活保障，营造良好的务工环境。

　　二是营造良好的人居环境。强化生态城市理念，将吴都文化、自然山水与现代文明融为一体，打造具有鄂州特色的城市品牌。开展群众性的植树造林活动和绿化美化活动，提高绿化覆盖率和城市人均公共绿地面积；加快城市森林建设，提高城市对环境的缓冲能力、自净能力和抗逆能力。着眼于市民生活质量和幸福指数，不断提高城市燃气普及率和供水普及率；建立完善廉租住房制度，逐步解决人均居住建筑面积10平方米以下的最低收入家庭住房问题；改善住房供应结构，建立适应高、中、低档不同层次需求的住房供应体系；全面建立乡镇及非公经济单位住房公积金制度，改善职工住房条件；按照简化方便、公开透明的原则，对与市民生活密切相关的政务流程进行系统优化，减少办事环节，缩短办事时限，提高生活便捷程度。

　　三是营造良好的治安环境。扎实开展“平安鄂州”创建活动，把社会治安综合治理措施落实到基层。继续进行企业、校园周边环境专项治理，重点打击强讨恶要、强装强卸、强揽工程等破坏企业生产经营和群众生活秩序的违法行为；加强文化市场整顿，加大网吧治理力度，为青少年健康成长创造良好的社会环境；对重点企业、重大项目继续实行民警驻勤制度，对侵害投资者和企业合法权益的行为进行严厉打击，对危害一方的黑恶势力始终保持高压态势，确保刑事案件、治安案件、集体上访发生率整体下降，确保政治安全、社会安全和信访安全。

　　（八）坚持不懈地推进统筹发展，努力建设和谐鄂州

　　一是扎实开展“四城”创建工作。争创国家园林城市、国家卫生城市、国家环境保护模范城市、全国创建文明城市工作先进城市，不断提高市民绿化意识、卫生意识、环保意识和文明意识，增强城市知名度和美誉度。深入开展以“八荣八耻”为主要内容的社会主义荣辱观教育，加强社会公德、职业道德、家庭美德建设，积极开展文化、科技、医疗卫生三下乡活动。认真实施“五五普法”，加强法制宣传，不断提高市民法律素质。积极开展对弱势群体的司法救助和法律援助工作。积极倡导扶贫济困、乐善好施的中华传统美德，大力表彰见义勇为和慈善义举，营造互相关爱的和谐生活氛围。

　　二是加快社会事业发展。整合教育资源，结合农村寄宿制学校建设，对农村学校进行布局调整。大力发展中职教育，支持鄂州职业大学发展。加强公共卫生体系建设，注重职业病防治和精神卫生，整合城乡医疗卫生资源，设立“惠民”医疗窗口，建设社区卫生服务中心。完善计划生育奖励扶助制度，加强对流动人口的计划生育管理和服务，开展出生人口性别比失衡问题的综合治理，稳定低生育水平。加强公共文化设施尤其是农村文化设施建设，进一步繁荣文化艺术、广播电视事业。广泛开展群众性体育活动，提高竞技运动水平。

　　三是加强就业和社会保障工作。强化政府促进就业职能，逐步建立市场导向的就业机制和困难群体就业帮扶机制；多渠道开发就业岗位，大力发展就业容量大的产业，确保城镇新增就业2万人以上。完善城镇职工基本养老、医疗和失业、工伤、生育保险制度，扩大社会保险覆盖面，提高各类所有制企业的参保率；完善城镇个体工商户和灵活就业人员的参保办法，研究制定适合农民工特点的社会保障措施，逐步做实个人账户；完善城市低保制度，努力做到动态管理下的应保尽保；加快城乡特殊困难群众社会救助体系建设，高度重视失地农民社会保障问题；完善农村“五保户”供养、低保户救助、灾民救济等制度，增加救助投入并适当提高救助标准。

　　四是高度重视安全生产和应急管理工作。落实安全生产责任制，加强群众安全知识宣传教育。强化安全生产监督管理，预防和减少各类事故。加大安全生产事故责任追究和处罚力度，严肃查处安全生产领域的失职渎职和腐败行为。加强工商行政管理、质量技术监督和食品、药品、烟草市场管理，保护消费者权益。开展风险隐患普查和治理工作，落实综合防范和处置措施，实行动态管理和监控。加强突发公共事件的信息报告和预警工作，提高应对突发公共事件的能力。

　　五是认真做好民族宗教、外事侨务等工作。全面贯彻党的民族政策，依法管理宗教事务。发挥台办、外事侨务等部门的职能作用，扩大对外交往与合作。重视人口老龄化问题，加大老龄事业投入力度。关心和支持青年、妇女、儿童事业发展，提高残疾人康复、教育、就业水平。认真做好物价、统计、邮政、石油石化、防震减灾、档案、方志、人防、消防、扶贫开发、农机、气象和国防教育、民兵建设等工作。

　　（九）坚持不懈地办好“十件实事”，解决群众最关心、最直接、最现实的利益问题

　　新的一年，我们将遵循“扶困、惠民、尽力”的原则，从群众最关心、最直接、最现实的利益问题入手，继续为全市人民办好“十件实事”：1、建立城乡一体的医疗救助体系。筹资3235万元，建立全市统筹的新型农村合作医疗制度，确保农村人口受益面达80%以上；筹资400万元，建立城镇居民医疗保险制度和社会救助机制，重点解决城镇低保人员住院医疗保险，逐步解决不同人群的医疗保障问题。2、启动对困难家庭的住房保障制度。筹资150万元，对1500户城市困难家庭实施租房补贴和租金核减；筹资300万元，实施“无房新建、危房改造”工程，解决农村300户特困家庭的住房困难。3、解决新区居民子女上学难问题。筹资4000万元，在城南新区新建一所初级中学；逐步提高城市周边村办小学的办学水平。4、改善农村学校的学习、生活环境。筹资1000万元，以“三建两改”（建宿舍、建食堂、建沼气池；改水、改厕）为重点，加快农村学校特别是寄宿制学校的新、改建工程。5、全面实行农村义务教育“两免一补”。筹资2850万元，全部免除农村义务教育阶段学生学杂费，并对贫困家庭的学生免费提供教科书，对贫困家庭的寄宿学生提供生活补助。6、继续实施安全饮水工程。筹资3120万元，解决8万农村人口安全饮水问题；筹资2100万元，对老城区、矿区1.1万户居民实施供水“户表改造工程”。7、继续实施农民体育健身工程。筹资300万元，在100个行政村实施农村体育健身设施建设。8、新建综合性城市福利院。筹资1000万元，新建综合性城市福利院（含流浪未成年人救助保护中心、儿童社会福利机构），对城市“三无”人员（无生活来源、无劳动能力、无法定赡养和抚养人）、流浪未成年人及弃婴实行集中供养。9、实施渡口建设规范达标。筹资460万元，对41处渡口进行改造，让群众过上安全渡。10、援助困难群体就业。筹资650万元，开发公益性岗位300个，对1500名城镇下岗职工再就业实行培训、职介和社保补贴；筹资160万元，对8000名农村劳动力进行转移就业培训。

　　三、加强政府自身建设

　　贯彻落实科学发展观、建设和谐鄂州和武汉城市圈发展最快的中等城市，对政府工作提出了新的要求；人民群众日益增长的物质文化生活需求，对政府工作提出了新的挑战；只有不断加强政府自身建设，切实提高行政力、执行力、落实力，才能适应新形势、开创新局面、再上新台阶。

　　（一）用心做事。聚精会神搞建设，一心一意谋发展是对政府工作人员的时代要求。一是努力建设学习型政府。开展创学习型机关，做人民满意的公务员活动。制定周密的学习计划，建立严格的考核办法，形成制度，常抓不懈。以培养一支思想开放、熟悉政策、通晓经济、精通业务、自觉奉献的公务员队伍为目标，制定政策和措施鼓励公务员加强政策理论和业务知识的学习，不断提高履行职责的本领。选拔一批优秀干部到沿海地区和大型企业挂职锻炼，不断提高发展经济的能力；聘请全国知名学者、专家举办专题讲座和与有关高校开展高素质人才培训合作，不断提高依法行政和处理复杂事务的能力。二是努力建设服务型政府。大力倡导基层优先和企业优先，努力形成“重商、亲商、安商”的政风和社会氛围，以便民、惠民为原则，提高行政服务中心效能。对重大项目实行代办制，市直各职能部门都要设立公共服务窗口，为企业和群众提供便捷高效的服务。三是努力建设务实型政府。实干兴业，空谈误事。各级政府工作人员要真正把心思用在干事创业上，把功夫下在狠抓落实上。要力戒浮躁、力戒虚夸、力戒空谈，勇于正视问题，勇于直面矛盾，勇于攻坚克难。每年确定二到三项在全省有特色有影响的工作和造福于企业和市民的措施，以人一我十、人十我百的拼劲和韧劲，团结一心，雷厉风行，以实实在在的工作业绩报效人民。

　　（二）公正待人。公平和正义是政府公信力的体现，是建设和谐社会的基石。一是坚持法律和制度面前人人平等。遵循职权法定的原则，严格界定各级政府和部门的职权，加强对行政许可、行政检查、行政征收、行政处罚等具体行政行为的监督，依法规范行政权力的运作程序。加强制度建设，进一步完善政府议事规则，规范政府的办事程序和方式，规范行政执法行为，形成科学决策、依法行政，靠制度管理、按规定办事的行政执行体系。依照法律精神和公正原则，切实解决好涉及人民群众切身利益问题。二是坚持保障人民群众的知情权、参与权。建立和健全民主决策机制、社会公示和听证制度、新闻发布制度、群众建议征集制度、社情民意反映制度，提高政府的科学决策和民主决策水平，让人民群众广泛享有知情权、参与权、监督权。加大政务事务公开力度，加强电子政务建设，打造“阳光行政”、“透明政府”，使政府与群众沟通的渠道更加畅通。三是坚持平等对待不同的社会群体。尊重外地人，尊重农民工，尊重社会弱势群体。注意听取和解决不同阶层的利益诉求。统筹协调各方面的利益关系，减少矛盾和纠纷的产生。在谋发展、搞建设时，综合考虑群众的长远利益和现实利益。加快公共财政建设，把有限的资金更多地用于解决低收入阶层的困难和问题。

　　（三）发展为先。发展是硬道理，是解决当前各种矛盾和问题的基础。一是要增强加快发展的能力。努力建设武汉城市圈发展最快的中等城市，首要的是建设一个最具行政效率的政府，关键是培养一支熟悉经济、勇于奉献的队伍，重点是建立和健全稳定、规范、透明、高效的工作机制，有计划、有秩序快速推进各项工作。二是增强安全发展的能力。以人为本首先是以人的生命为本，科学发展首先要安全发展。坚持经济社会发展与环境保护、生态建设、安全发展相统一，既讲求经济效益，也要重视社会效益和生态效益。将安全发展与经济发展和社会进步同步规划、同步实施、同步发展。进一步畅通信访渠道，落实信访工作责任，形成有效的利益协调机制、诉求表述机制、矛盾调处机制、权益保障机制。三是增强可持续发展的能力。统筹考虑经济增长与资源支撑和要素保障的关系，不仅要安排好当前的发展，还要为未来的发展创造更好条件，把主要精力放在事关全局和长远发展的“打基础、管长远”的工作上，坚决反对短期行为，用实实在在的工作为鄂州人民谋利益。

　　（四）人民至上。政府是人民的政府，必须以人民利益为最高利益。一是正确运用权力。政府的权力来自人民，一切权力属于人民。必须牢固树立正确的权力观，坚持权为民所用，情为民所系，利为民所谋，必须大力加强廉政建设，不断完善制度，做到民主决策、科学决策。自觉抵制拜金主义、享乐主义和极端个人主义，建立健全教育、制度、监督并重的体系，坚决查处以权谋私的腐败分子，注重从源头上防治腐败。二是自觉接受监督。建立健全行政执法责任制度、绩效考核制度，完善政府的监督与考核体系。自觉接受人大的法律监督和工作监督，认真执行人大及其常委会的决议决定；自觉接受政协民主监督，坚持重大事项与人民政协协商制度；自觉接受人民群众监督，认真办理人大代表建议和政协委员提案，逐步养成在监督下办事的习惯，努力建设人民满意的政府。三是真心实意为群众办事。把为人民群众办实事作为政府义不容辞的责任和义务，关注民生，尊重民意，体察民情，认真解决人民群众反映的切身利益问题，注重从人民群众的柴米油盐、衣食住行、安危冷暖等方面入手，及时解决人民群众最关心、最直接、最现实的利益问题，坚决纠正损害群众利益的行为。

　　各位代表，鄂州是一方充满希望的热土，加快发展的前景光明，潜力巨大。在新的一年里，我们一定要倍加珍惜上下一心的良好局面，倍加珍惜蓬勃旺盛的发展势头，在市委的正确领导下，紧紧依靠和团结全市人民，全面落实科学发展观，以更加昂扬的斗志、更加饱满的热情、更加务实的作风，又好又快地完成各项目标任务，为建设武汉城市圈发展最快的中等城市而努力奋斗！
